# The effect of comorbidity on the use of adjuvant chemotherapy and type of regimen for curatively resected stage III colon cancer patients

**DOI:** 10.1002/cam4.632

**Published:** 2016-01-15

**Authors:** Mei‐Chin Hsieh, Trevor Thompson, Xiao‐Cheng Wu, Timothy Styles, Mary B. O'Flarity, Cyllene R. Morris, Vivien W. Chen

**Affiliations:** ^1^ Louisiana Tumor Registry and Epidemiology ProgramSchool of Public HealthLouisiana State University Health Sciences CenterNew OrleansLouisiana; ^2^ Cancer Surveillance BranchDivision of Cancer Prevention and ControlNational Center for Chronic Disease Prevention and Health PromotionCenters for Disease Control and PreventionAtlantaGeorgia; ^3^ Public Health InstituteCalifornia Cancer RegistrySacramentoCalifornia

**Keywords:** Adjuvant chemotherapy, chemotherapy regimen, colon cancer, comorbidity, treatment

## Abstract

Postsurgical chemotherapy is guideline‐recommended therapy for stage III colon cancer patients. Factors associated with patients not receiving adjuvant chemotherapy were identified in numerous studies; comorbidity was recognized as an important factor besides patient's age. We assessed the association between comorbidity and the use of adjuvant chemotherapy and type of chemotherapy regimen. Stage III colon cancer patients who underwent surgical resection were obtained from ten Centers for Disease Control and Prevention (CDC)‐NPCR Specialized Registries which participated in the Comparative Effectiveness Research (CER) project. Comorbidity was classified into no comorbidity recorded, Charlson, non‐Charlson comorbidities, number, and severity of Charlson comorbidity. Pearson chi‐square test and multivariable logistic regression were employed. Of 3180 resected stage III colon cancer patients, 64% received adjuvant chemotherapy. After adjusting for patient's demographic and tumor characteristics, there were no significant differences in receipt of chemotherapy between Charlson and non‐Charlson comorbidity. However, patients who had two or more Charlson comorbidities or had moderate to severe disease were significantly less likely to have chemotherapy (ORs 0.69 [95% CI, 0.51–0.92] and 0.62 [95% CI, 0.42–0.91], respectively) when compared with those with non‐Charlson comorbidity. In addition, those with moderate or severe comorbidities were more likely to receive single chemotherapy agent (*P* < 0.0001). Capecitabine and FOLFOX were the most common single‐ and multi‐agent regimens regardless of type of comorbidity grouping. Both the number and severity of comorbidity were significantly associated with receipt of guideline‐recommended chemotherapy and type of agent in stage III resected colon cancer patients. Better personalized care based on individual patient's condition ought to be recognized.

## Introduction

Clinical trials have proved that postoperative chemotherapy with fluorouracil‐based regimens significantly reduced the risk of tumor recurrence and improved survival for both young and old stage III colon cancer patients 1, 2, 3, 4, 5, 6, 7. Adjuvant chemotherapy has been a guideline‐recommended treatment for resected stage III colon cancer patients for over two decades 8. Although several chemotherapy regimens have demonstrated their safety and efficacy of use in elderly colorectal cancer patients 5, 9, the utilization of adjuvant chemotherapy still occurs less frequent in elderly patients, particularly when comorbidities are present 10, 11, 12. Unfortunately, the median age at diagnosis for colon cancer patients is 70% and 40% of cases occurred in patients aged 75 and older 13. In addition, elderly cancer patients are more likely to have noncancer chronic diseases; an average of 4.2 comorbid conditions occurred in patients aged ≥75 compared to an average of 2.9 among those aged 55–64 14. Furthermore, patient's concurrent comorbidity, besides age, is a key factor that influences physicians' decision of not recommending adjuvant chemotherapy to stage III colon cancer patients because of concerning toxicity and life‐expectancy 15, 16, 17.

The main objective of this study was to examine the association between comorbid conditions and receiving adjuvant chemotherapy for stage III colon cancer patients using ten population‐based cancer registries' data. The association was assessed by the type, the number, and the severity of comorbid conditions. We also evaluated whether comorbid conditions affect the selection of chemotherapy regimen for those receiving adjuvant chemotherapy. Finally, we examined the reasons for not receiving guideline‐recommended adjuvant chemotherapy.

## Patients and Methods

Colon cancer patients diagnosed in 2011 were obtained from ten population‐based cancer registries that were the Centers for Disease Control and Prevention (CDC)'s National Program of Cancer Registries (NPCR) Specialized Registries and participated in the Comparative Effectiveness Research (CER). These states were Alaska, Colorado, Idaho, Louisiana, New Hampshire, North Carolina, Rhode Islands, and Texas, as well as 13 counties of the Sacramento region in California and five metro counties in Florida which covered about 27% of the United States population with very high representation among minority populations 18. As part of the CER project, detailed first course of cancer‐directed treatment and comorbidities were collected.

### Study cohort

The data included in this study were restricted to stage III colon cancer patients who underwent surgical resection. We selected colon cancer cases based on the International Classification of Diseases for Oncology 3rd edition (ICD‐O‐3) topographic codes: C180, C182‐C189 (Appendix was excluded, *n* = 51) and morphologic codes: 8000–8152, 8154–8231, 8243–8245, 8247–8248, 8250–8576, 8940–8950, 8980–8981. Derived 7th Edition AJCC staging, which was originated from the directly coded Collaborative Staging version 02.05 19, was used to select colon cancer with stage III disease. Patients who died within 30 days of colon resection (*n* = 134), had missing information on adjuvant chemotherapy (*n* = 428), health insurance, residential, poverty status, grade, or unknown number of positive lymph nodes (*n* = 210) were excluded from this study.

### Description of variables

#### Adjuvant chemotherapy and chemotherapy agents

The main outcome of interest was whether stage III colon cancer patients received chemotherapy after surgical resection. For chemotherapy drugs, we categorized them into single agent, multiple agents and unknown. Single agents included 5‐fluorouracil (5‐FU), capecitabine, irinotecan and oxaliplatin. Multiple agents included FOLFOX (5‐FU and oxaliplatin), FOLFIRI (5‐FU and irinotecan), FOLFOXIRI (5‐FU, oxaliplatin, Irinotecan,), XELOX (capecitabine and oxaliplatin), and XELIRI (capecitabine and irinotecan). Irinotecan is a key component of first/second‐line treatment regimens for metastatic colorectal cancer 20 and is not the standard adjuvant chemotherapy for resected stage III colon cancer patients. However, a study showed that stage III colon cancer patients with positive CIMP (CpG island methylator phenotype) benefited from fluorouracil with Irinotecan by improving overall survival 21. Therefore, irinotecan has been chosen as an alternative chemotherapy agent to treat stage III colon cancer patients. Regimens that contain 5‐FU but substituted Floxuridine, which is an analogy drug of 5‐FU, were categorized to the same regimen.

#### Comorbidity classification

Comorbid conditions included pre‐existing health conditions and/or those diagnosed during the cancer treatment but excluded conditions related to a previous cancer diagnosis. Coding instructions were based on the Facility Oncology Registry Data Standards (FORDS) manual 22. Comorbid conditions were coded according to the ICD‐9 clinical modification (ICD‐9‐CM) diagnosis codes and included the following codes: 00100‐13980, 24000–99990, E8700‐E8799, E9300‐E9499, V0720‐V0739, V1000‐V1590, V2220‐ V2310, V2540, V4400‐V4589, and V5041‐V5049. We used the coding algorithms developed by Quan 23 to define comorbidities in ICD‐9‐CM which was the enhanced version of the Deyo's adaption of Charlson Index 24. Fifteen diseases from the Charlson Comorbidities list were identified and included in this study.

The comorbid conditions were categorized based on the comorbidity type (no comorbidity recorded, non‐Charlson comorbidity, Charlson comorbidity), the number of Charlson comorbidity (one comorbidity and two or more comorbidities), and the severity of Charlson comorbidity (mild and moderate to severe). No comorbidity recorded by the CER specialized cancer registries included those where no comorbidity was documented in the medical chart or unknown whether patient had a coexisting condition when cancer diagnosed. Non‐Charlson comorbidity included other conditions that were not listed in the Charlson Comorbidities list. We used the Charlson comorbidity weights to determine the severity of comorbidity and it was categorized into: mild (condition with weight = 1) and moderate to severe (weight = 2, 3, and 6). Because patients with moderate and/or severe comorbidities are often considered for the toxicity, life‐expectancy and side effect of chemotherapy 15 and only nine patients had severe comorbidity, we combined patients with moderate or with severe disease into one group in the analysis. The moderate to severe diseases includes diabetes with chronic complication, hemiplegia, renal disease, moderate to severe liver disease, and AIDS.

#### Classification of patient and tumor characteristics

Health insurance, indicating the patient's insured status at diagnosis and during the first course of treatment, was grouped into no insurance, private (including Medicare with private supplement), Medicaid, and Medicare and other public insurance. Census track poverty level and census tract residence were obtained from the United States Census Bureau. We categorized census track poverty into <10%, 10–20%, and >20% and census tract residence into 100% urban, 100% rural, and mixed. Other covariates included in the analysis were patient's age (<50, 50–59, 60–69, 70–79, ≥80), sex, race/ethnicity (non‐Hispanic white, non‐Hispanic black, Hispanic, and non‐Hispanic other), state of residence, tumor stage (IIIA, IIIB, IIIC), histological grade, number of positive lymph nodes (<6, 6–11, and ≥12), and whether colon cancer was the first primary cancer (Yes, No).

### Statistical analysis

The proportions of comorbidity and type of disease were calculated for each age group. The frequencies of chemotherapy agent/regimen were cross‐tabulated with comorbid condition. Pearson chi‐square test was used to determine statistical differences in bivariate analysis. Logistic regression was used to examine the association between comorbidity and adjuvant chemotherapy for both unadjusted and adjusted models. Three multivariable models based on the method of grouping comorbid conditions were analyzed to assess the association between comorbidity and receiving adjuvant chemotherapy after controlling for patient and tumor characteristics.

Comorbidity is commonly grouped by the number of Charlson diseases in cancer research. In order to thoroughly examine the association between comorbid conditions and receiving adjuvant chemotherapy, we assessed the association by the type of comorbidity, the number of comorbidities, and the severity of comorbid conditions. Three models were used to assess these comorbidity groupings. Model I assessed the effect by type of comorbid condition, model II by the number of Charlson comorbid diseases, and model III by the severity of disease. Reason for not receiving and reason for not completing chemotherapy were assessed across three comorbidity groups as well.

## Results

A total of 3180 eligible stage III colon cancer patients diagnosed in 2011 were included in the data analysis. There were slightly more males (50.8%) then females, predominantly non‐Hispanic whites (66%) and about 46% of them were aged 70 and older. Patients aged 70–79 years old had the highest percentage of Charlson comorbidity followed by aged 80 and older, 37.2% versus 35.7%, respectively (Table 1); over one‐third of them had two or more Charlson comorbidities (34.0% vs. 37.7%, respectively). In contrast, non‐Charlson comorbidity occurred more frequently in younger age groups and gradually decreased as age increased. The percentages of no comorbidity recorded were evenly distributed across all age groups, around 30%. Diabetes without complications was the most common Charlson comorbid condition for all ages combined followed by chronic pulmonary disease (Table 1). Furthermore, mild liver disease was more prevalent in age <50 and congestive heart failure was pronouncedly higher in age ≥80.

**Table 1:** Percent distributions of comorbidity grouping and type of Charlson comorbidity for stage III colon cancer patients by age group

Comorbidity grouping	Age at diagnosis					
	<50 (*N* = 350)	50–59 (*N* = 543)	60–69 (*N* = 837)	70–79 (*N* = 766)	≥80 (*N* = 684)	Total (*N* = 3180)
No comorbidity recorded	29.7	29.8	29.9	29.0	31.9	30.1
Non‐Charlson comorbidity	53.4	46.6	40.0	33.8	32.5	39.5
Charlson comorbidity	16.9	23.6	30.1	37.2	35.7	30.4
	*N* a ^* *^ *= 59*	*N* a ^* *^ *= 128*	*N* a ^* *^ *= 252*	*N* a ^* *^ *= 285*	*N* a ^* *^ *= 244*	*N * a ^* *^ *=968*
By number						
1	91.5	77.3	71.8	66.0	62.3	69.6
2+	8.5	22.7	28.2	34.0	37.7	30.4
By severity						
Mild (weight = 1)	89.8	84.4	86.9	84.6	80.7	84.5
Moderate to severe (weight = 2,3,6)	10.2	15.6	13.1	15.4	19.3	15.5
By type of Charlson comorbidity	*N* b ^* *^ *= 66*	*N* b ^* *^ *= 165*	*N* b ^* *^ *= 339*	*N* b ^* *^ *= 403*	*N* b ^* *^ *= 361*	*N* b ^* *^ *= 1334*
Myocardial infarction	3.0	3.6	5.6	7.7	9.4	6.9
Congestive heart failure	4.5	6.1	9.1	10.7	18.6	11.5
Peripheral vascular disease	0.0	3.6	4.1	5.0	6.9	4.9
Cerebrovascular disease	1.5	2.4	3.8	3.0	3.3	3.1
Dementia	0.0	0.0	0.3	0.7	3.0	1.1
Chronic pulmonary disease	22.7	12.1	18.0	23.3	19.4	19.5
Rheumatic disease	1.5	1.8	1.5	1.2	0.3	1.1
Peptic ulcer disease	3.0	1.2	1.8	1.5	1.9	1.7
Mild liver disease	16.7	12.7	7.1	3.0	1.9	5.6
Diabetes without Complications	37.9	43.0	38.9	33.0	21.6	32.9
Hemiplegia/paraplegia	0.0	1.2	0.3	1.0	0.3	0.6
Diabetes with complications	0.0	3.0	2.7	0.5	2.2	1.8
Renal disease	4.5	6.1	4.7	8.7	10.8	7.7
Moderate/severe liver disease	1.5	2.4	0.9	0.5	0.0	0.7
AIDS	3.0	0.6	1.2	0.2	0.3	0.7

a Number of colon cancer patients with at least one Charlson comorbidity.

b Total number of Charlson comorbidities.

Overall, 64% of colon cancer patients with resected stage III disease received adjuvant chemotherapy. The percent receiving adjuvant chemotherapy declined from 86% in patients aged <50 years to only 28% in those aged ≥80 years (Table 2). The percentage of patients receiving adjuvant chemotherapy varied by state of residence as well, ranging from 55% in Texas to 79% in Louisiana. Additionally, 69% of patients having non‐Charlson comorbidity received adjuvant chemotherapy compared to only 51% of those with two or more and 49% with moderate to severe Charlson comorbidity.

**Table 2:** Percentages, odds ratios, and 95% confidence intervals (CI) of receiving adjuvant chemotherapy for stage III colon cancer patients based on different comorbidity groupings

Variables	*N* (% Received chemotherapy)*N* = 3180 (64.3%)	Unadjusted Odds Ratio (95% CI)	Adjusted Odds Ratio (95% CI)		
			Model I	Model II	Model III
Comorbidity type					
No comorbidity recorded	956 (62.7)	0.76 (0.64–0.91)	0.86 (0.68–1.08)	–	–
Non‐Charlson comorbidity	1256 (68.9)	ref	ref	–	–
Charlson comorbidity	968 (60.1)	0.68 (0.57–0.81)	0.89 (0.73–1.09)	–	–
Number of Charlson Comorbidity					
No comorbidity recorded	956 (62.7)	0.76 (0.64–0.91)	–	0.86 (0.68–1.08)	–
Non‐Charlson comorbidity	1256 (68.9)	ref	–	ref	–
1 Charlson comorbidity	674 (64.2)	0.81 (0.67–0.99)	–	1.01 (0.80–1.26)	–
2 + Charlson comorbidities	294 (50.7)	0.46 (0.36–0.60)	–	0.69 (0.51–0.92)	–
Severity of Charlson Comorbidity	–	** **–	–	–	–
No comorbidity recorded	956 (62.7)	0.76 (0.64–0.91)	–	–	0.86 (0.68‐1.08)
Non‐Charlson comorbidity	1256 (68.9)	ref	–	–	ref
Mild (weight = 1)	818 (62.2)	0.75 (0.62–0.90)	–	–	0.96 (0.77–1.19)
Moderate to severe (weight = 2,3,6)	150 (48.7)	0.43 (0.31–0.60)	–	–	0.62 (0.42–0.91)
Age at diagnosis					
<50	350 (86.3)	ref	ref	ref	ref
50–59	543 (79.9)	0.63 (0.44–0.92)	0.62 (0.42–0.91)	0.62 (0.42–0.91)	0.62 (0.42–0.91)
60–69	837 (74.8)	0.47 (0.34–0.66)	0.46 (0.32–0.66)	0.47 (0.32–0.67)	0.46 (0.32–0.67)
70–79	766 (64.1)	0.28 (0.20–0.40)	0.28 (0.19–0.40)	0.28 (0.20–0.41)	0.28 (0.19–0.41)
≥80	684 (28.2)	0.06 (0.04–0.09)	0.06 (0.04–0.08)	0.06 (0.04–0.09)	0.06 (0.04–0.08)
Sex					
Male	1616 (66.6)	ref	ref	ref	ref
Female	1564 (62.0)	0.82 (0.71–0.94)	0.98 (0.83–1.16)	0.97 (0.82–1.15)	0.97 (0.82–1.15)
Race/ethnicity					
Non‐hispanic white	2097 (63.3)	ref	ref	ref	ref
Non‐hispanic black	487 (74.3)	1.68 (1.34–2.09)	1.29 (0.99–1.69)	1.29 (0.98–1.68)	1.31 (1.00–1.72)
Hispanic	499 (59.7)	0.86 (0.70–1.05)	1.01 (0.79–1.30)	1.01 (0.78–1.30)	1.02 (0.79–1.31)
Non‐hispanic other	97 (59.8)	0.86 (0.57–1.31)	0.74 (0.46–1.20)	0.73 (0.45–1.18)	0.73 (0.45–1.18)
Health insurance					
No insurance	165 (73.3)	1.04 (0.72–1.49)	0.81 (0.55–1.21)	0.81 (0.54–1.20)	0.81 (0.54–1.20)
Private	1377 (72.6)	ref	ref	ref	ref
Medicaid	345 (57.4)	0.51 (0.40–0.65)	0.48 (0.36–0.64)	0.48 (0.36–0.64)	0.48 (0.36–0.65)
Medicare and other public insurance	1293 (56.2)	0.48 (0.41–0.57)	0.81 (0.66–0.98)	0.80 (0.66–0.98)	0.80 (0.66–0.98)
Census tract residence					
100% Urban	1843 (60.8)	ref	ref	ref	ref
100% Rural	280 (67.1)	1.32 (1.01–1.72)	1.04 (0.76–1.43)	1.03 (0.75–1.42)	1.04 (0.75–1.42)
Mixed	1057 (69.7)	1.48 (1.26–1.74)	1.30 (1.06–1.59)	1.30 (1.06–1.59)	1.29 (1.05–1.58)
Census track poverty					
Poverty <10%	1721 (63.7)	ref	ref	ref	ref
Poverty 10–20%	916 (67.5)	1.18 (1.00–1.40)	1.12 (0.92–1.36)	1.11 (0.91–1.35)	1.12 (0.92–1.36)
Poverty > 20%	543 (61.0)	0.89 (0.73–1.08)	0.82 (0.64–1.04)	0.82 (0.64–1.04)	0.82 (0.64–1.05)
State of residence					
AK	34 (70.6)	2.00 (0.95–4.22)	1.91 (0.81–4.53)	1.96 (0.82–4.67)	1.93 (0.81–4.60)
CA	195 (69.2)	1.87 (1.35–2.60)	2.82 (1.87–4.24)	2.80 (1.86–4.22)	2.82 (1.87–4.24)
CO	269 (69.9)	1.93 (1.45–2.58)	2.78 (2.00–3.87)	2.79 (2.00–3.89)	2.81 (2.01–3.91)
FL	564 (58.2)	1.16 (0.94–1.42)	1.88 (1.42–2.48)	1.86 (1.41–2.46)	1.86 (1.41–2.46)
ID	77 (74.0)	2.37 (1.40–4.01)	2.70 (1.50–4.85)	2.69 (1.50–4.85)	2.70 (1.50–4.85)
LA	328 (78.7)	3.07 (2.29–4.10)	3.57 (2.55–5.00)	3.55 (2.54–4.97)	3.51 (2.51–4.91)
NC	546 (74.9)	2.48 (1.98–3.12)	3.01 (2.30–3.94)	3.00 (2.29–3.93)	3.01 (2.30–3.94)
NH	87 (63.2)	1.43 (0.91–2.25)	1.91 (1.13–3.21)	1.92 (1.14–3.25)	1.94 (1.15–3.28)
RI	54 (59.3)	1.21 (0.69–2.11)	2.17 (1.15–4.10)	2.19 (1.16–4.14)	2.21 (1.17–4.17)
TX	1026 (54.6)	ref	ref	ref	ref
AJCC stage III					
IIIA	364 (59.3)	ref	ref	ref	ref
IIIB	2070 (65.0)	1.27 (1.01–1.60)	1.57 (1.21–2.04)	1.58 (1.21–2.05)	1.58 (1.21–2.05)
IIIC	746 (64.9)	1.27 (0.98–1.64)	1.57 (1.11–2.24)	1.59 (1.12–2.26)	1.58 (1.11–2.24)
Grade					
Well/moderately differentiated	2321 (65.4)	ref	ref	ref	ref
Poor/undifferentiated	859 (61.4)	0.84 (0.71–0.99)	0.99 (0.82–1.21)	0.99 (0.82–1.21)	1.00 (0.82–1.21)
First primary					
Yes	2592 (66.0)	ref	ref	ref	ref
No	588 (56.8)	0.68 (0.56–0.81)	0.90 (0.73–1.12)	0.91 (0.74–1.13)	0.91 (0.74–1.13)
Number of positive LN					
<6	2546 (64.0)	ref		ref	ref
6–11	467 (66.6)	1.12 (0.91–1.38)	1.09 (0.81–1.46)	1.10 (0.82–1.48)	1.08 (0.81–1.45)
≥12	167 (62.9)	0.95 (0.69–1.32)	0.89 (0.58–1.37)	0.88 (0.57–1.36)	0.87 (0.57–1.35)

The effect of different comorbidity groupings after adjusting for patient and tumor characteristics is presented in Table 2. Before adjustment, stage III colon cancer patients without comorbidity recorded or with Charlson comorbid condition(s) were less likely to receive chemotherapy when compared with those with non‐Charlson comorbid condition(s) after surgical resection (OR, 0.76 [95% CI, 0.64–0.91] and OR, 0.68 [95% CI, 0.57–0.81], respectively). These statistically significant associations no longer existed after adjusting for patient and tumor characteristics in model I (Table 2). However, statistically significant differences were observed when examined by number of Charlson comorbidity and by severity of disease. Patients who had two or more Charlson comorbidities (Model II) or who had moderate to severe disease (Model III) were less likely to receive adjuvant chemotherapy than those with non‐Charlson comorbidity, OR = 0.69 (95% CI, 0.51–0.92) and OR = 0.62 (95% CI, 0.42–0.91), respectively.

The likelihood of receiving postoperative chemotherapy decreased with advancing age in all adjusted models (Table 2). In addition, patients who had Medicaid, Medicare or other public health insurance were less likely to receive adjuvant chemotherapy than those with private health insurance; the results were consistent across all three models (Table 2). In contrast, patients who resided in urban‐rural mixed areas or had more advanced stage of disease at diagnosis had higher likelihood of receiving adjuvant chemotherapy than their counterparts. We did not observe any racial/ethnic variations in the multivariable analysis, except Model III with marginal significance. Model III showed that non‐Hispanic blacks had 31% (95% CI, 1.00–1.72) higher odds of receiving adjuvant chemotherapy than non‐Hispanic whites. Male, lower tumor grade, and colon cancer as the first primary were statistically significantly associated with receiving adjuvant chemotherapy in the univariate analysis; however, these significant associations were not observed after adjusting for all predictors.

**Figure 1:**
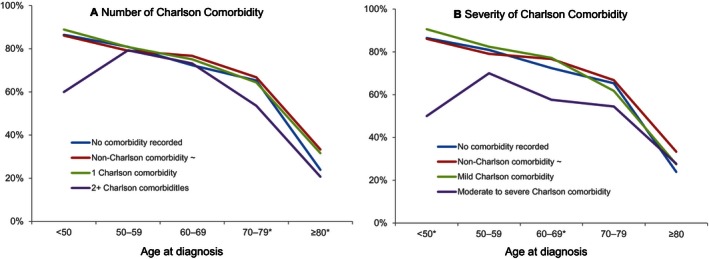
Proportion of patients with stage III colon cancer receiving adjuvant chemotherapy by comorbidity grouping and age group.~Non‐Charlson comorbidity as reference. *At least one group is statistically significant when comparing with the reference group.

To better understand the relationship between the comorbid condition and receiving adjuvant chemotherapy for stage III colon cancer patients, we examined the percentage of those receiving adjuvant chemotherapy among groupings of comorbidity stratified by age group. The results are presented in Figure 1. Severity of Charlson comorbidity displayed more variation than number of Charlson comorbidity on receiving adjuvant chemotherapy among five age groups. The percentages of patients receiving adjuvant chemotherapy were similar between patients with one Charlson comorbidity and those with non‐Charlson comorbidity (Fig. 1A). However, compared to patients with non‐Charlson comorbidity group, patients with two or more Charlson comorbidities had significantly lower percent receipt of adjuvant chemotherapy in age 70–79 and age ≥80 (Fig. 1A). Likewise patients with moderate to severe Charlson comorbidity were less likely to receive adjuvant chemotherapy in age <50 and age 60–69 (Fig. 1B). There were no statistical differences when comparing patients with one Charlson comorbidity or with mild Charlson comorbidity to those with non‐Charlson comorbidity.

We also examined the relationship between the type of chemotherapy agent and comorbidity grouping and noted statistically significant association between comorbidity and the type of chemotherapy regimen (*P *<* *0.0001). Overall, 21% of stage III colon cancer patients received single chemotherapy agents, 54% received multiple agents and 25% of patients had unknown type of chemotherapy regimen. About 62% of patients without comorbidity recorded and 55% with non‐Charlson comorbidity received multiple chemotherapy agents, compared to only 29% of patients with moderate to severe Charlson comorbid condition (Table 3). The most commonly used single agent and multiple agents across all groupings of comorbid conditions were capecitabine and FOLFOX.

**Table 3:** Percentages of chemotherapy agents for stage III colon cancer patients by comorbidity grouping

Chemotherapy Regimen	No comorbidity recorded	Non‐Charlson comorbidity	Charlson comorbidity	Number of Charlson comorbidity		Severity of Charlson comorbidity	
	(*N* = 655)	(*N* = 914)	(*N* = 607)	1 (*N* = 448)	2 + (*N* = 159)	Mild (N = 528)	Moderate to severe (*N* = 79)
Single agent	*16.6*	*21.3*	*26.7*	*23.7*	*35.2*	*25.4*	*35.4*
Fluorouracil	5.3	6.7	7.6	7.1	8.8	8.3	2.5
Capecitabine	7.3	8.3	11.5	10.5	14.5	10.0	21.5
Irinotecan	0.2	0.1	0.0	0.0	0.0	0.0	0.0
Oxaliplatin	3.5	5.8	7.2	5.8	11.3	6.6	11.4
Other single agent	0.3	0.4	0.3	0.2	0.6	0.4	0.0
Multiple agents	*61.5*	*54.7*	*45.3*	*47.5*	*39.0*	*47.7*	*29.1*
FOLFOX	52.4	41.7	36.1	37.7	31.4	38.6	19.0
XELOX	5.0	7.4	5.6	6.9	1.9	6.1	2.5
FOLFIRI	0.3	0.9	1.0	0.9	1.3	0.8	2.5
XELIRI	0.2	0.0	0.0	0.0	0.0	0.0	0.0
FOLFOXIRI	0.3	0.4	0.0	0.0	0.0	0.0	0.0
Other multiple agents	3.4	4.3	2.6	2.0	4.4	2.3	5.1
Unknown	*21.8*	*24.0*	*28.0*	*28.8*	*25.8*	*26.9*	*35.4*

**Figure 2:**
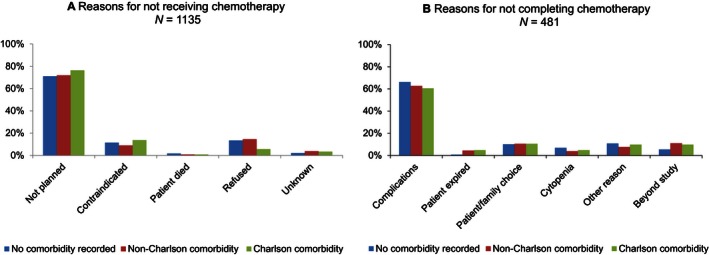
Reason for not receiving or not completing adjuvant chemotherapy for stage III colon cancer patients by comorbidity type.

Figure 2 shows the reason for not receiving and not completing adjuvant chemotherapy by comorbidity type. The major reason for not following the guideline‐recommended chemotherapy among stage III colon cancer patients was that adjuvant chemotherapy was not planned for the first course of treatment regardless the comorbidity type (Fig. 2A), ranging from 71–76%. Of the 2046 patients receiving adjuvant chemotherapy, over half (*n* = 1095) of patients had unknown completion status. For those patients not completing the planned chemotherapy with known reason (*n* = 481), developing complication was the main reason across all three types (Fig. 2B), especially for patients without comorbidity recorded (66%).

## Discussion

In this study, we found 7 out of 10 stage III colon cancer patients had at least one recorded comorbid condition. This result was consistent with a previous study 25. Of those patients (*n* = 2224) with comorbid condition(s), 43.5% had at least one Charlson comorbidity which was more pronounced in age ≥70. Similar with previous reports 9, 10, 11, our study also revealed that age remains as a strong predictor for not receiving guideline‐recommended adjuvant chemotherapy for patients with stage III colon cancer. Compared with patients aged <50, the ORs of receiving adjuvant chemotherapy declined dramatically from 0.63 in age group 50–59 to 0.06 in age group ≥80. However, these ORs remained similar after adjusting for comorbidity and other predictors in all three models. We observed that the number and severity of comorbidity had a substantially negative association with guideline‐recommended adjuvant chemotherapy for stage III colon cancer patients which was consistent with previous studies 9, 10, 11, 25. Patients with moderate to severe Charlson comorbidity and those with two or more Charlson comorbidities had lower odds of receiving adjuvant chemotherapy (OR = 0.62 and OR = 0.69 respectively) compared to those with non‐Charlson comorbidity after adjusting for age and other predictors.

Literature has shown patient life‐expectancy and potential toxicity were the main concerns of physicians in administering postoperative chemotherapy in stage III colon cancer patients 15, 16. These survey studies examined the preferences of surgeons versus oncologists and found that both age and severity of comorbidity affected the surgeon's decision for not referring to an oncologist and oncologist's decision for not recommending adjuvant chemotherapy to stage III colon cancer patients 15, 16. In particular, the oncologist's decision was more profoundly influenced by patient's comorbid condition 16. Studies demonstrated that life‐expectancy of colorectal cancer patients substantially declined with the increasing number of coexisting chronic diseases 26, 27, 28. Differences in chemotherapy drug toxicity between younger and older stage III colon cancer patients were well established in previous studies 5, 14, 29. Older patients received the same benefit as their younger counterparts from fluorouracil‐based adjuvant therapy without a significant increase in toxicity;^5^ whereas the oxaliplatin with FOLFOX regimen showed a slightly higher rate of cytopenia among older patients 14. However, the association between the toxic effects of adjuvant chemotherapy and type of comorbidity was not clear for stage III colon cancer patients. Cassidy et al. 30 reported that age was not related to increasing toxicity in metastatic colorectal cancer patients treated with first‐line oral capecitabine after controlling for any possible renal function side effects. In addition, certain Charlson comorbidities including chronic obstructive pulmonary disease, congestive heart failure, peptic ulcer disease, renal disease, and AIDS were significantly associated with an increasing risk of chemotherapy‐induced febrile neutropenia in cancer patients 31.

A prospective observational cohort study demonstrated that treatment modification due to low‐grade toxicity occurred more often in cancer patients with multiple comorbidities, but age was not significantly associated with it 32. We observed that patients with either two or more comorbidities or with moderate to severe Charlson comorbidity were less likely to receive FOLFOX, a multiple agent regimen. Instead, physicians tended to treat those patients with capecitabine or oxaliplatin which has a favorable safety profile compared with intravenous 5‐fluorouracil/leucovorin 30. In addition, for patients who received adjuvant chemotherapy, treatment complication was the primary reason for the early termination.

Our study has several strengths. The study cohort includes ten population‐based cancer registries which cover about 27% of the United States population and has a high proportion of minority populations 18. Although first course treatment is collected by all population‐based cancer registries as part of routine abstracting, it has been known that adjuvant therapy data are incomplete, especially those administrated in medical oncology offices. Therefore, the CER registries were provided additional resources to collect all treatment data items required by the CER project. Thus, CER data have much more complete first course treatment including detailed chemotherapy regimens and dosages which are not routinely collected in population‐based cancer registries.

Limitations of this study should be noted. Because of the shift of medical practice, the majority of chemotherapy was administrated in medical oncology offices. For states with large territories, the collection and completeness of chemotherapy information has been challenging. Coding of comorbid conditions were based on the FORDS coding instructions 22. However, not all coexisting diseases listed in ICD‐9‐CM were considered “valid” comorbidities for abstract collection. For example, a coexisting cancer, which may alter the treatment plan would not have been included. Nevertheless, our analysis had taken into account whether the colon cancer was the first primary cancer or not thus minimizing the impact of any coexisting cancer. Also patients without comorbidities or with unknown comorbidity were categorized into the same group as no comorbidity recorded by the CER specialized cancer registries according to the FORDS coding manual, thus we were not able to compare Charlson or Non‐Charlson comorbidity group with the known “no comorbidity” group. Intestinal perforation, obstruction or stoma is known to impact the use of adjuvant chemotherapy;^11^ however, this information was not collected by CER registries. Furthermore, our study data did not collect the functional limitations and geriatric syndromes which are known to influence physician treatment decisions in addition to comorbidity 33.

In conclusion, comorbidities may limit treatment options due to the increasing toxicity of specific chemotherapy agents and reduction in life‐expectancy. As the number or severity of comorbidities increased, the likelihood of receiving guideline‐concordant chemotherapy treatment decreased comparatively in stage III colon cancer patients, particularly among the elderly. Given that increasing age is paralleled by an increase in comorbid conditions, an enhanced personalized practice that takes into account the cancer patient's coexisting chronic illnesses should be the focus in order to improve the quality of cancer care as emphasized in the 2009 Annual Meeting of the American Society of Clinical Oncology 34.

## Conflict of Interest

The authors declare no financial conflicts of interest in this work. The findings and conclusions in this article are those of the authors and do not necessarily represent the official position of the Centers for Disease Control and Prevention.

## References

[1] Moertel, C. G. , T. R. Fleming , J. S. Macdonald , et al. 1995 Fluorouracil plus levamisole as effective adjuvant therapy after resection of stage III colon carcinoma: a final report. Ann. Intern. Med. 122:321–326. 7847642 10.7326/0003-4819-122-5-199503010-00001

[2] O'Connell, M. J. , J. A. Mailliard , M. J. Kahn , et al. 1997 Controlled trial of fluorouracil and low‐dose leucovorin given for 6 months as postoperative adjuvant therapy for colon cancer. J. Clin. Oncol. 15:246–250. 8996149 10.1200/JCO.1997.15.1.246

[3] Gill, S. , C. L. Loprinzi , D. J. Sargent , et al. 2004 Pooled analysis of fluorouracil‐based adjuvant therapy for stage II and III colon cancer: who benefits and by how much? J. Clin. Oncol. 22:1806–1979. 10.1200/JCO.2004.09.059 15067028

[4] Andre, T. , C. Boni , L. Mounedji‐Boudiaf , et al. 2004 Oxaliplatin, fluorouracil, and leucovorin as adjuvant treatment for colon cancer. N. Engl. J. Med. 350:2343–2351. 15175436 10.1056/NEJMoa032709

[5] Sargent, D. J. , R. M. Goldberg , S. D. Jacobson , et al. 2001 A pooled analysis of adjuvant chemotherapy for resected colon cancer in elderly patients. N. Engl. J. Med. 345:1091–1097. 11596588 10.1056/NEJMoa010957

[6] Iwashyna, T. J. , and E. B. Lamont . 2002 Effectiveness of adjuvant fluorouracil in clinical practice: a population‐based cohort study of elderly patients with stage III colon cancer. J. Clin. Oncol. 20:3992–3998. 12351596 10.1200/JCO.2002.03.083

[7] Sundararajan, V. , N. Mitra , J. S. Jacobson , et al. 2002 Survival associated with 5‐fluorouracil‐based adjuvant chemotherapy among elderly patients with node‐positive colon cancer. Ann. Intern. Med. 136:349–357. 11874307 10.7326/0003-4819-136-5-200203050-00007

[8] NIH Consensus Conference . 1990 Adjuvant therapy for patients with colon and rectal cancer. JAMA 264:1444–1450. 2202842

[9] Goldberg, R. M. , I. Tabah‐Fisch , H. Bleiberg , et al. 2006 Pooled analysis of safety and efficacy of oxaliplatin plus fluorouracil/leucovorin administered bimonthly in elderly patients with colorectal cancer. J. Clin. Oncol. 24:4085–4091. 16943526 10.1200/JCO.2006.06.9039

[10] Schrag, D. , and L. D. Cramer . 2001 BachPB, Begg CB. Age and adjuvant chemotherapy use after surgery for stage III colon cancer. J. Natl Cancer Inst. 93:850–857. 11390534 10.1093/jnci/93.11.850

[11] Janssen‐Heijnen, M. L. G. , S. Houterman , V. E. P. P. Lemmens , et al. 2005 Prognostic impact of increasing age and co‐morbidity in cancer patients: a population‐based approach. Crit. Rev. Oncol./Hematol. 5:231–240. 10.1016/j.critrevonc.2005.04.008 15979890

[12] Hsieh, M. C. , Y. W. Chiu , C. Velasco , et al. 2013 Impact of race/ethnicity and socioeconomic status on adjuvant chemotherapy use among elderly patients with stage III colon cancer. J. Registry Manage. 40:180–187. 24625772

[13] H. , N. , Noone A. M., Krapcho M. et al. (eds). 1975‐2011. SEER Cancer Statistics Review, National Cancer Institute. Bethesda, MD,Available at: http://seer.cancer.gov/csr/1975_2011/, based on November 2013 SEER data submission, posted to the SEER web site, April 2014.

[14] Yancik, R. 1997 Cancer burden in the aged: an epidemiologic and demographic overview. Cancer 20:1273–1283. 9317180

[15] Keating, N. L. , M. B. Landrum , C. N. Klabunde , et al. 2008 Adjuvant chemotherapy for stage III colon cancer: do physicians agree about the importance of patient age and comorbidity? J. Clin. Oncol. 26:2532–2537. 18487570 10.1200/JCO.2007.15.9434

[16] Krzyzanowska, M. K. , M. M. Regan , M. Powell , et al. 2009 Impact of patient age and comorbidity on surgeon versus oncologist preferences for adjuvant chemotherapy for stage III colon cancer. J. Am. Coll. Surg. 208:202–209. 19228531 10.1016/j.jamcollsurg.2008.10.016

[17] Shayeb, M. E. , A. Scarfe , Y. Yasui , et al. 2012 Reason physicians do not recommend and patients refuse adjuvant chemotherapy for stage III colon cancer: a population based chart review. BioMed Res. Notes 5:269. 10.1186/1756-0500-5-269 PMC3405463 22676354

[18] Chen, V. W. , C. R. Eheman , C. J. Johnson , et al. 2014 Enhancing Cancer Registry Data for Comparative Effectiveness Research (CER) Project: overview and Methodology. J. Registry Manage. 41:103–112. PMC4524450 25419602

[19] Collaborative Stage Data Collection System . 2014 Version 02.05. Available at: https://cancerstaging.org/cstage/schema/Pages/version0205.aspx. (accessed June 16, 2014).

[20] Fuchs, C. , E. P. Mitchell , and P. M. Hoff . 2006 Irinotecan in the treatment of colorectal cancer. Cancer Treat. Rev. 32:491–503. 16959432 10.1016/j.ctrv.2006.07.001

[21] Shiovitz, S. , M. M. Bertagnolli , L. A. Renfro , et al. 2014 CpG island methylator phenotype is associated with response to adjuvant irinotecan‐based therapy for stage III colon cancer. Gastroenterology 47:637–645. 24859205 10.1053/j.gastro.2014.05.009 PMC4143495

[22] Facility Oncology Registry Data Standards (FORD) manual .2014. Available at: https://www.facs.org/quality-programs/cancer/ncdb/registrymanuals/cocmanuals/fordsmanual. (accessed June 16, 2014).

[23] Quan, H. , V. Sundararajan , P. Halfon , et al. 2005 Coding algorithms for defining comorbidities in ICD‐9‐CM and ICD‐10 administrative data. Med. Care 43:1130–1139. 16224307 10.1097/01.mlr.0000182534.19832.83

[24] Deyo, R. A. , D. C. Cherkin , and M. A. Ciol . 1992 Adapting a clinical comorbidity index for use with ICD‐9‐CM administrative databases. J. Clin. Epidemiol. 45:613–619. 1607900 10.1016/0895-4356(92)90133-8

[25] Sarfati, D. , S. Hill , T. Blakely , et al. 2009 The effect of comorbidity on the use of adjuvant chemotherapy and survival from colon cancer: a retrospective cohort study. BMC Cancer 9:116. doi:10.1186/1477‐2407‐9‐116. 19379520 10.1186/1471-2407-9-116 PMC2678274

[26] Yancik, R. , P. A. Ganz , C. G. Varricchio , and B. Conley . 2001 Perspectives on comorbidity and cancer in older patients: approaches to expand the knowledge base. J. Clin. Oncol. 19:1147–1151. 11181680 10.1200/JCO.2001.19.4.1147

[27] Ouellette, J. R. , D. G. Small , and P. M. Termuhlen . 2004 Evaluation of Charlson‐Age Comorbidity Index as predictors of morbidity and mortality in patients with colorectal carcinoma. J Gastrointest Surg. 8:1061–1067. 15585394 10.1016/j.gassur.2004.09.045

[28] Gross, C. P. , G. J. McAvay , H. M. Krumholz , et al. 2006 The effect of age and chronic illness on life expectancy after a diagnosis of colorectal cancer: implications for screening. Ann. Intern. Med. 145:646–653. 17088577 10.7326/0003-4819-145-9-200611070-00006

[29] McCleary, N. J. , E. Dotan , and I. Browner . 2014 Refining the chemotherapy approach for older patients with colon cancer. L Clin Oncol. 32:2570–2580. 10.1200/JCO.2014.55.1960 25071118

[30] Cassidy, J. , C. Twelves , E. Van Cutsem , et al. 2002 First‐line oral capecitabine therapy in metastatic colorectal cancer: a favorable safety profile compared with intravenous 5‐fluorouracil/leucovorin. Ann. Oncol. 13:566–575. 12056707 10.1093/annonc/mdf089

[31] Chao, C. , J. H. Page , S. J. Yang , et al. 2014 History of chronic comorbidity and risk of chemotherapy‐induced febrile neutropenia in cancer patients not receiving G‐CSF prophylaxis. Ann. Oncol. 25:1821–1829. 24915871 10.1093/annonc/mdu203

[32] Kalsi , T. , Babic‐Illman G., Fields P., et al. 2014 The impact of low‐grade toxicity in older people with cancer undergoing chemotherapy. BJC 111:2224–2228. doi: 10.1038/bjc.2014.496. 25268369 10.1038/bjc.2014.496 PMC4264435

[33] Koroukian, S. M. , F. Xu , P. M. Bakaki , et al. 2010 Comorbidities, functional limitations, and geriatric syndromes in relation to treatment and survival patterns among elders with colorectal cancer. J. Gerontol. A Biol. Sci. Med. Sci. 65A:322–329. 20018824 10.1093/gerona/glp180 PMC2904594

[34] Schilsky, R. L. 2009 Personalizing cancer care: American Society of Clinical Oncology presidential address 2009. J. Clin. Oncol. 27:3725–3730. 19581526 10.1200/JCO.2009.24.6827

